# Treatment of Multiple Sclerosis With Teriflunomide. Multicenter Study of Real Clinical Practice in the Valencian Community-Spain

**DOI:** 10.3389/fneur.2021.727586

**Published:** 2021-10-29

**Authors:** Lamberto Landete, Francisco Pérez-Miralles, Sara García, Antonio Belenguer, Francisco Gascón, Jose Andres Domínguez, María Carcelén-Gadea, Carlos Quintanilla-Bordás, Laura Navarro, Laura Gabaldón, Bonaventura Casanova

**Affiliations:** ^1^Neurology Service, Hospital Universitario Dr. Peset, Valencia, Spain; ^2^Centro, Servicio y Unidad de Referencia, Neuroinmunology Unit, Hospital Universitari i Politécnic La Fe, Valencia, Spain; ^3^Neurology Service, Hospital General Universitario de Castellón, Castellón de la Plana, Spain; ^4^Demyelinating Diseases Unit, Hospital Clínico Universitario de Valencia, Valencia, Spain; ^5^Neurology Service, Hospital General de Valencia, Valencia, Spain; ^6^Neurology Service, Hospital Universitario de Elche, Elche, Spain; ^7^Neurology Service, Hospital Comarcal de Gandía, Valencia, Spain

**Keywords:** teriflunomide, MS therapy, real world evidence (RWE), cohort analysis, multicentric study

## Abstract

**Introduction:** We have different treatment alternatives for relapsing-remitting multiple sclerosis–RRMS–within the so-called platform drugs. It would be desirable to know the ideal drug for each patient. Real clinical practice studies provide us with data on drug efficacy in the medium and long term, safety beyond clinical trials, and can help us to know the patient profile appropriate for each therapy.

**Material and Methods:** An observational multicenter study of real clinical practice in patients with RRMS who were treated with teriflunomide in the Valencian Community, since teriflunomide was authorized in Spain. The database created for this study collects retrospectively patients followed prospectively in the MS clinics.

**Objectives:** To analyze the efficacy and safety of teriflunomide treatment in patients with RRMS under the conditions of real clinical practice, and to identify a patient profile responding to the treatment.

**Results:** We obtained data from 340 patients who received at least one dose of 14 mg teriflunomide. The patients were 69.4% female to 30.6% male, had a mean age of 46.4 years, and a mean time of progression of MS of 11.5 years. The mean pre-teriflunomide relapse rate was 0.4 years, the mean EDSS scorewas 1.98, IgG Oligoclonal bands were present in the CSF of 66.2% of the patients, IgM Oligoclonal bands were present in 46.9%, and the mean number of gadolinium-enhancing lesions was 1.07 lesions per patient at the beginning of treatment. The average number of treatments previously received was 1.04, and 28.53% were naïve. After a follow-up of up to 4 years, a reduction in the annualized and cumulative annualized relapse rate was observed in the first year, in the second year, and in the third year, compared to the pre-treatment year. The EDSS scores were stabilized throughout the follow-up. Likewise, there was a reduction in gadolinium-enhancing lesions in the 1st and 2nd years compared to the pre-treatment period. Applying different generalized multiple linear regression models, we identified a profile of a responding patient to teriflunomide as a male without IgM oligoclonal bands in the CSF, a previous EDSS score of <3, and more than 5 years duration of MS.

## Introduction

Since January 2015, there are two new oral drugs available in Spain for the initial treatment of relapsing-remitting multiple sclerosis: teriflunomide and dimethyl fumarate. Teriflunomide has demonstrated efficacy vs placebo in two phase 3 (1, 2) trials, in both clinical and MRI variables.

When oral drugs were authorized, a proportion of the patients treated with interferon or glatiramer acetate switched to oral therapies: some for reasons related to efficacy, others due to adverse events, and a large part for reasons related to the comfort or convenience of the oral route of administration.

When the patients switched to oral therapies, the following questions were raised. In those patients whose symptoms were well-controlled with injectables for long periods of time, would MS remain well-controlled when switching to an oral treatment? What would the long-term safety profile of oral therapies look like? What is the persistence of these drugs? Would the efficacy data from the trials be reproduced in real clinical practice?

Real clinical practice studies provide us with data on efficacy in the medium and long term, safety beyond clinical trials, and may help us to know the patient profile appropriate for each therapy. Real clinical practice studies aim to answer these questions, providing information on long-term efficacy, new or unexpected adverse events, and other data about some MS aspects like persistence or adherence.

## Materials and Methods

This is an observational, post-authorization, real clinical practice study. We analyze patients diagnosed with RRMS who were treated with teriflunomide in seven monographic multiple sclerosis clinics from hospitals in the public network of the Valencian Community on the Spanish Mediterranean coast. The database created for this study collected patients who were followed prospectively.

### Objectives

To analyze the efficacy and safety of treatment with teriflunomide in patients with RRMS under the conditions of real clinical practice, and to identify a profile of a patient who responds well to teriflunomide.

Efficacy is measured by the evolution of the annual relapse rate in year 1, year 2, year 3, and year 4 of follow-up, compared to the annual relapse rate in the pretreatment year. Likewise, the mean number of relapses accumulated throughout the follow-up period, compared to the number of relapses in the year prior to the start of the teriflunomide treatment, has also been evaluated. Other efficacy variables are as follows: the evolution of disability as measured by the Kurtzke Expanded Disability Status Scale (EDSS) taking as a reference the EDSS score before the start of teriflunomide treatment and the evolution of gadolinium-enhancing lesions in MRI.

A relapse was defined as any new neurological symptom not associated with fever or infection lasting for at least 24 h and accompanied by new neurological signs. Disability progression has been considered as any increase in EDSS score, regardless of its magnitude. It would be more accurate to call it *worsening of disability*.

The statistical analysis was performed using R software (version 4.0.2). First, a descriptive analysis of the demographic and clinical characteristics of the patients was performed. The categorical variables were described by absolute and relative frequencies. Quantitative variables were summarized by their mean, standard deviation, median, and interquartile range.

The Friedman test was used to assess whether there were significant changes in the clinical quantitative variables of patients prior to and during treatment. When significant differences were found, the paired Wilcoxon test was used to identify the pairs of follow-up moments between which these differences were found. In order to reduce the possibility of false positives due to multiple pairwise comparisons, *p*-values were adjusted using the method of Benjamini and Hochberg (3).

Subsequently, the number of relapses suffered by patients at different times of follow-up after the start of treatment was compared according to their demographic and clinical characteristics prior to the start of treatment. Non-parametric Mann-Whitney and Kruskal-Wallis tests were used to compare the number of relapses according to the categorical variables.

A variety of generalized multiple linear regression models were constructed to evaluate the response to treatment of the patients based on their demographic and clinical characteristics prior to the start of treatment. Specifically, a Poisson regression model was used to analyze the number of accumulated relapses that patients presented during treatment. Since the number of accumulated relapses may be influenced by the follow-up time of the patient, the follow-up time was considered as an offset of the number of relapses in the model. Additionally, logistic regression models were used to evaluate some dichotomous variables related to the response to treatment: the presence or absence of relapses at some point during treatment, an increase or decrease in EDSS score after the start of treatment, a value on the EDSS greater (or equal or less) than 3.5 at some point during the observation period, the presence or absence of Gd lesions, and the continuation or not of treatment. The explanatory variables considered in the models were age, sex, relapses in the year before treatment (no, yes), Gd lesions (no, yes), IgM oligoclonal bands (absent, present), T2 lesions (<10, ≥10), EDSS score at diagnosis, disease progression time (<5, ≥5 years), and previous treatment (no, yes). The quality of the fit of the Poisson regression model was evaluated from the *deviance* and the Chi-square goodness-of-fit test. The quality of the fit of the logistic regression models was evaluated using the Hosmer-Lemeshow test and exploration of the residuals (verifying that there were no observations with large residuals).

## Results

### Demographic and Clinical Baseline Characteristics

We obtained data from 340 patients with RRMS, who had received at least one dose of 14 mg teriflunomide daily.

Of these, 236 (69.4%) are women and 104 (30.60%) are men. The mean age was 46.44 years (13–65 years). The time of evolution of MS from the onset of symptoms was 138.24 months (median 120 months) or 11.52 years (median 10 years) (Table 1).

**Table 1:** Baseline characteristics of the studied population.

**Demographic variable**	***N* = 340**
**Age**	
Mean (DT)	46.44 (10.24)
Median (Q1, Q3)	46.5 (40, 53)
**Sex**	
Female, *n* (%)	236 (69.41%)
Male, *n* (%)	104 (30.59 %)
**Evolution time (months)**	
Mean (DT)	138.24 (108.7)
Median (Q1, Q3)	120 (48, 200.5)
**Evolution time (years)**	
Mean (DT)	11.52 (9.06)
Median (Q1, Q3)	10 (4, 16.71)
**Pre-teriflunomide clinical features**	
**Relapses 2 years earlier**	
Mean (DT)	0.46 (0.75)
Median (Q1, Q3)	0 (0, 1)
0, *n* (%)	185 (65.6%)
1, *n* (%)	74 (26.24%)
≥2, *n* (%)	23 (8.16%)
**Relapses previous year**	
Mean (DT)	0.4 (0.58)
Median (Q1, Q3)	0 (0, 1)
0, *n* (%)	218 (64.31%)
1, *n* (%)	109 (32.15%)
≥2, *n* (%)	12 (3.54%)
**EDSS at diagnosis**	
Mean (DT)	1.9 (1.17)
Median (Q1, Q3)	2 (1, 2.5)
**EDSS pre-teriflunomide**	
Mean (DT)	1.8 (1.29)
Median (Q1, Q3)	2 (1, 2.5)
**IgG oligoclonal bands (CSF)**	
Absent *n* (%)	40 (11.94%)
Present *n* (%)	222 (66.27%)
**IgM oligoclonal bands (CSF)**	
Present *n* (%)	78 (53.06%)
Present *n* (%)	69 (46.94%)
**RM N°** **T2 lesions at diagnosis**	
0 lesions, *n* (%)	2 (0.86%)
1–4 lesios, *n* (%)	47 (20.17%)
5–9 lesios, *n* (%)	72 (30.9%)
10–20 lesios (%)	82 (35.19%)
>20 lesios, *n* (%)	30 (12.88%)
**RM Gd+** **lesions at diagnosis**	
Mean (DT)	0.82 (1.49)
Median (Q1, Q3)	0 (0, 1)
**RM N°** **T2 lesions pre-teriflunomide**	
0 lesions, *n* (%)	0 (0%)
1–4 lesions, *n* (%)	54 (19.85%)
5–9 lesions, *n* (%)	65 (23.9%)
10–20 lesions (%)	75 (27.57%)
>20 lesions, *n* (%)	43 (15.81%)
**RM Gd+** **lesions pre-teriflunomide**	
Mean (DT)	1.07 (8.39)
Median (Q1, Q3)	0 (0, 0)
**Treatments pre-Teriflunomide**	
**Number of previous treatments**	
Mean (DT)	1.04 (0.9)
Median (Q1, Q3)	1 (0, 1)
**Pre-treatment**	
None, *n* (%)	97 (28.53%)
Glatiramer acetate, *n* (%)	72 (21.18%)
Intramuscular interferon, *n* (%)	19 (5.59%)
Subcutaneous interferon, *n* (%)	111 (32.65%)
Fingolimod, *n* (%)	6 (1.76%)
Natalizumab, *n* (%)	1 (0.29%)
Azatioprina, *n* (%)	1 (0.29%)
Dimetilfumarate, *n* (%)	24 (7.06%)
Other, *n* (%)	9 (2.65%)
**Reason for the change to teriflunomide**	
Naïve, *n* (%)	96 (28.32%)
Inefficiency, *n* (%)	34 (10.03%)
Systemic adverse effects, *n* (%)	60 (17.70%)
Skin adverse effects, *n* (%)	82 (24.19%)
Analytical adverse effects, *n* (%)	19 (5.60%)
Patient's desire, *n* (%)	43 (12.68%)

The relapse rate two years prior to the start of treatment was 0.46 years. In the previous year, it was 0.4 years. The mean level of disability measured by EDSS score was 1.9 (median 2) at the time of diagnosis and 1.98 (median of 2) at the time of starting the teriflunomide treatment.

A total of 222 patients were carriers of IgG oligoclonal bands (66.27%), and 69 patients were carriers of IgM Oligoclonal bands in their CSF (46.94%).

Before the start of teriflunomide treatment, 54 patients (19.85%) had between 1 and 4 lesions in their T2 brain MRI, 65 patients (23.9%) had between 5 and 9 lesions, 75 patients (27.57%) had between 10 and 20 lesions, and 43 patients (15.81%) had more than 20 lesions in their T2 MRI sequences. The mean number of gadolinium-enhancing lesions was 1.07 lesions per patient.

The mean number of treatments received before the start of teriflunomide was 1.04 (median 1). In 97 patients (28.53%), teriflunomide was their first therapy, 72 (21.81%) patients were being treated with glatiramer acetate, 19 (5.59%) with intramuscular interferon, 111 (2.65%) with subcutaneous interferon, 6 (1.76%) with fingolimod, 1 (0.29%) with natalizumab, 1 (0.29%) with azathioprine, 24 (7.06%) dimethylfumarate, and 9 (2.65%) with other therapies. Of the patients from other therapies, the reason for the switch to teriflunomide was ineffectiveness in 34 (10.03%), systemic adverse effects in 60 (17.7%), skin adverse effects in 82 (24.19%), analytical adverse effects in 19 (5.6%), patient desire in 43 (12.68%), and other reasons in 13 patients (1.47%).

### Efficacy Results

**Figure 1:**
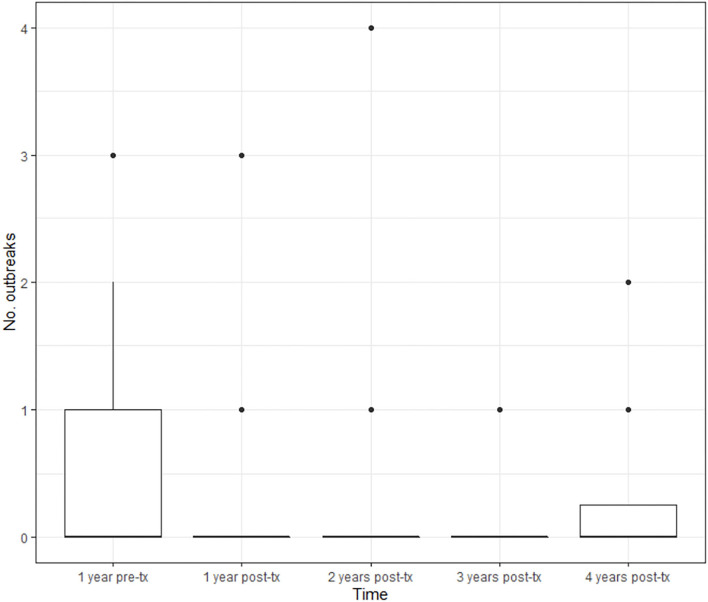
Annual evolution of the relapse rate. BOX, Interquartile range; Groos line, medias; Dots, Extrem values (>1.5 × interquartile range) disability.

**Figure 2:**
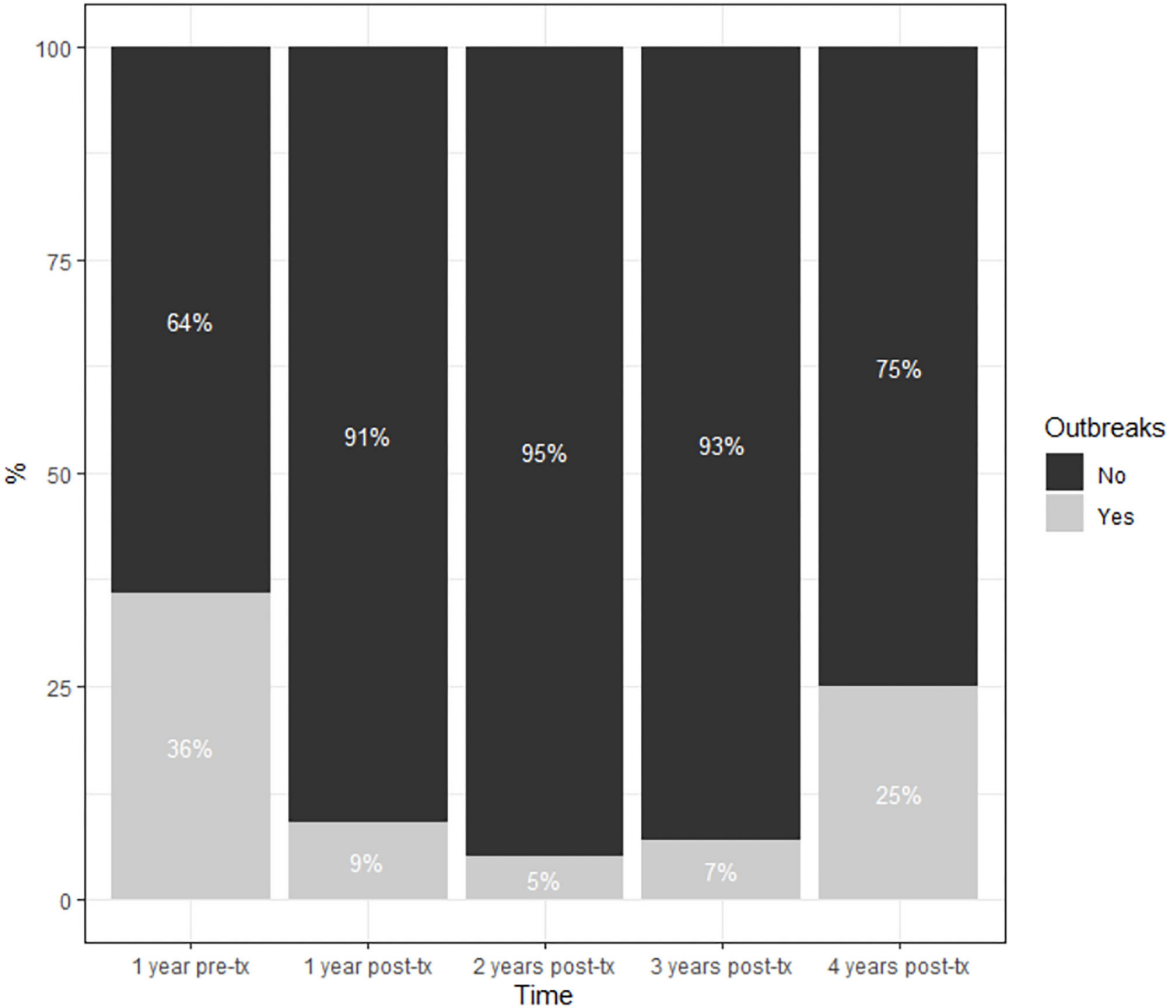
Evolution of patients free of relapses after the start of treatment with Teriflunomide.

Efficacy results are summarized in Table 2. There was a reduction in the annual relapse rate in the first year (mean value 0.1 vs. 0.4 relapses; *p* < 0.0001), the second year (mean 0.09 vs. 0.4; *p* < 0.0001), and at the third year (0.07 vs. 0.4; *p* = 0.0013), compared to the year before starting teriflunomide (Figure 1). Because there are only data from 8 patients with 4 years of follow-up, the comparison between the fourth year and the pre-treatment year is worthless. The mean cumulative relapse in the first 2 and 3 years was 0.17 relapses in both periods of time, and the comparison with the mean of relapses in the pre-teriflunomide year was statistically significant (*p* = 0.0001 and *p* = 0.0350, respectively). An increase in the number of patients free of relapses was observed throughout the follow-up (Figure 2).

**Table 2:** Results, clinical, and radiological evolution.

		**Year pre-terifl**	**1^**°**^ Year post-terifl**	**2^**°**^ year post-terifl**	**3^**°**^ year post-terifl**	**4^**°**^ year post-terifl**
N° relapse	*n*	339	236	162	54	8
	Mean (DT)	0.4 (0.58)	0.1 (0.34)	0.09 (0.48)	0.07 (0.26)	0.38 (0.74)
	Median (Q1, Q3)	0 (0, 1)	0 (0, 0)	0 (0, 0)	0 (0, 0)	0 (0, 0.25)
	P*		<0.0001	<0.0001	0.0013	–
N° relapse	*n*	339	236	162	54	8
Accumulated	Mean (DT)	0.4 (0.58)	0.1 (0.34)	0.17 (0.59)	0.17 (0.42)	0.38 (0.74)
	Median (Q1, Q3)	0 (0, 1)	0 (0, 0)	0 (0, 0)	0 (0, 0)	0 (0, 0.25)
	P**		<0.0001	0.0001	0.0350	–
EDSS	*n*	340	232	129	53	8
	Mean (DT)	1.98 (1.29)	2 (1.34)	1.99 (1.43)	2.09 (1.32)	2.25 (1.04)
	Median (Q1, Q3)	2 (1, 2.5)	2 (1, 2.5)	2 (1, 2.5)	2 (1, 2.5)	2.25 (1.38, 3.12)
	*P*		ns	ns	ns	–
Gd +	*n*	280	213	151	45	4
lesions	Mean (DT)	0.37 (1.27)	0.18 (1.1)	0.06 (0.26)	0.09 (0.36)	0 (0)
	Median (Q1, Q3)	0 (0, 0)	0 (0, 0)	0 (0, 0)	0 (0, 0)	0 (0, 0)
	*P*		0.0052	<0.0001	ns	–
T2	*n*	237	147	67	32	4
lesions	1–4 lesions	54 (22.7%)	7 (4.7%)	4 (5.9%)	1 (3.1%)	0 (0%)
	5–9 lesions	65 (27.4%)	40 (27.2%)	18 (11.9%)	7 (21.8%)	1 (25%)
	10–20 lesions	75 (31.6%)	52(35.3%)	27 (40.2%)	9 (28.1%)	3 (75%)
	<20 lesions	43 (18.1%)	48 (32.6%)	18 (26.8%)	11 (34.3%)	1 (0%)

*Comparison with the year prior to treatment with teriflunomide*.

*p ≤ 0.05. ns, not significant; DT, Standard deviation; Q1, First quartile; Q3, Third quartile*.

p^*^ Comparison of each year with respect to the pre-treatment year.

p^**^ Comparison of cumulative years with respect to the pre-treatment year.

**Figure 3:**
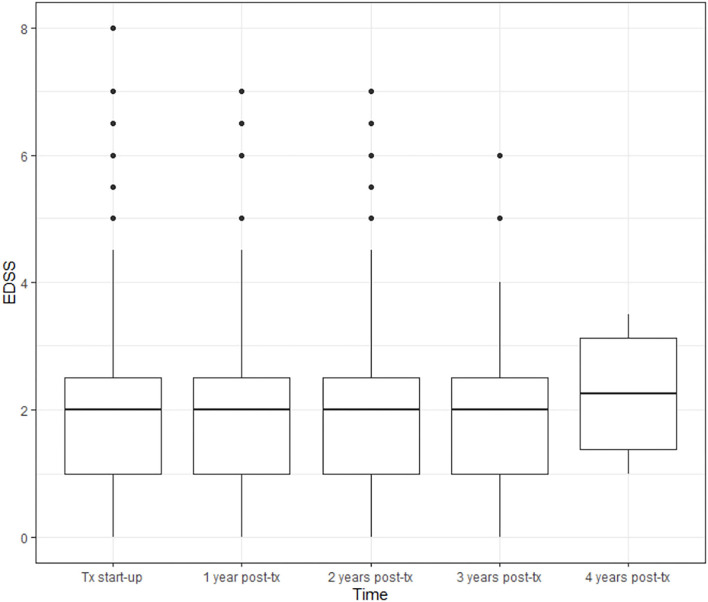
Evolution of disability as measured by EDSS throughout follow-up. BOX, Interquartile range; Groos line, medias; Dots, Extrem values (>1.5 × interquartile range) disability.

Throughout the follow-up, the patients' EDSS scores do not change significantly when compared to the score at baseline (Figure 3). The baseline mean EDSS score was 1.98. At the end of the first year of treatment, the mean EDSS score was 2, at the end of the second year it was 1,99, at the end of the 3rd year it was 2,09, and at the end of the 4th year it was 2.25 (*p* = 0.2397).

**Figure 4:**
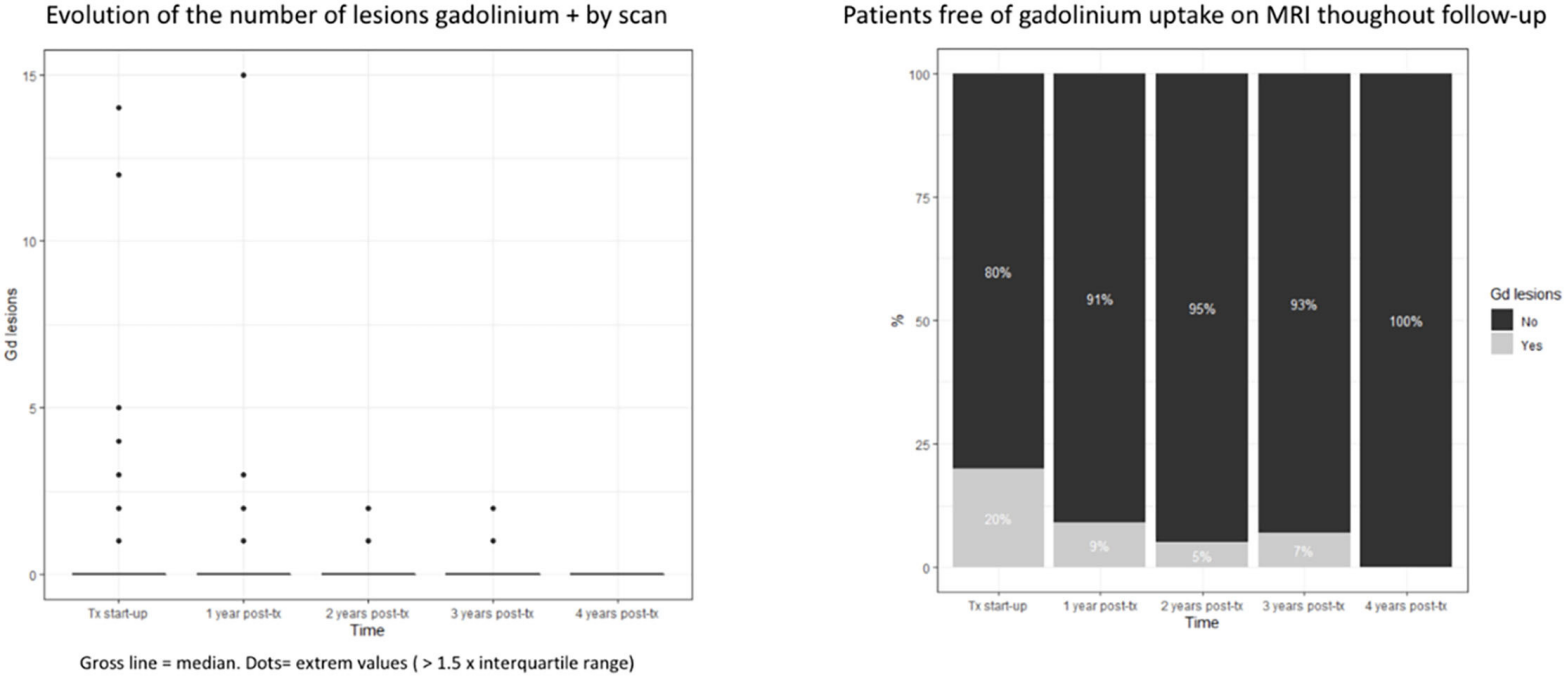
Evolution of the number of the RM gadolinium + lesions. BOX, Interquartile range; Groos line, medias; Dots, Extrem values (>1.5 × interquartile range) disability.

Regarding MRI measurements, we observed a reduction in the mean number of T1-enhancing lesions throughout the follow-up, comparing with the pre-treatment MRI; the mean was 0.37 lesions per patient. At the end of the first year, the mean number of Gd+ lesions was 0.18 (*p* = 0.0052), at the end of the second year it was 0.06 (*p* < 0.0001), and at the end of the 3rd and 4th years no significant differences were found (Figure 4).

To analyze the number of lesions in the T2 MRI sequences, we have grouped patients into intervals to minimize the bias derived from the fact that the lesions have been counted by the radiologist or the neurologist when this data has not been specified in the radiological reports. The MRI protocols used in the participants' centers may be different. Despite these limitations, it is observed that the percentage of patients in each range of the number of lesions remains relatively stable, although there is a trend of an increase in the group of patients with >20 lesions (non-significant differences).

When we tried to correlate the different clinical and demographic characteristics with the responses to treatment defined with the annual relapse rate in the second and third year (Supplementary Table 1), we found that there are no differences in the response to treatment if the patient's age is above or below 40 years in the first year (*p* = 0.8876), the second year (*p* = 0.6541), or the third year (*p* = 0.0442). We also found no differences according to sex throughout the follow-up (*p* = 0.5858 in year 1, *p* = 0.2318 in year 2, *p* = 0.9826 in year 3). Likewise, there are no differences in the number of relapses if the evolution lasts for more or <5 years (*p* = 0.0611 in the 1st year, *p* = 0.6351 in the second year, *p*= 0.7118 in the 3rd year). We have seen that there are significant differences in the first year in favor of those patients who did not suffer relapses in the previous year (*p* = 0.0250); these differences are lost in the second year (*p* = 0.3874) and in the 3rd year (*p* = 0.0719). We also observed no difference between patients who had an EDSS score above or below 3 (*p* = 0.8414 in year 1, *p* = 0.3827 in year 2, and *p* = 0.2555 in year 3).

The presence of IgG oligoclonal bands was not correlated with a greater or lesser frequency of relapses in year 1 (*p* = 0.3069), year 2 (*p* = 0.3521), or year 3 (*p* = 0.3933). The presence of IgM oligoclonal bands was correlated with a better response to treatment in the first year in those patients with negative IgM oligoclonal bands (*p* = 0.0261). This significance was lost in the second year (*p* = 0.4016) and in the third year (*p* = 0.4595).

There are no differences in the frequency of relapses between patients with more or <10 lesions in T2 in the first year (*p* = 0.0728), the second year (*p* = 0.0762), or the third year (*P* = 0.8658). We found differences in the frequency of relapses in the first year with fewer relapses in those patients without gadolinium-enhancing lesions before starting treatment with teriflunomide (*p* = 0.0050). This significance was lost in the second year (*p* = 0.4106) and in the 3rd year (*p* = 0.7497).

There was a significantly lower frequency of relapses in the first year in those patients who came from other treatments compared to untreated patients (*p* = 0.0216). These significant differences were lost in the second year (*p* = 0.7817) and in the third year (*p* = 0.6637).

**Figure 5:**
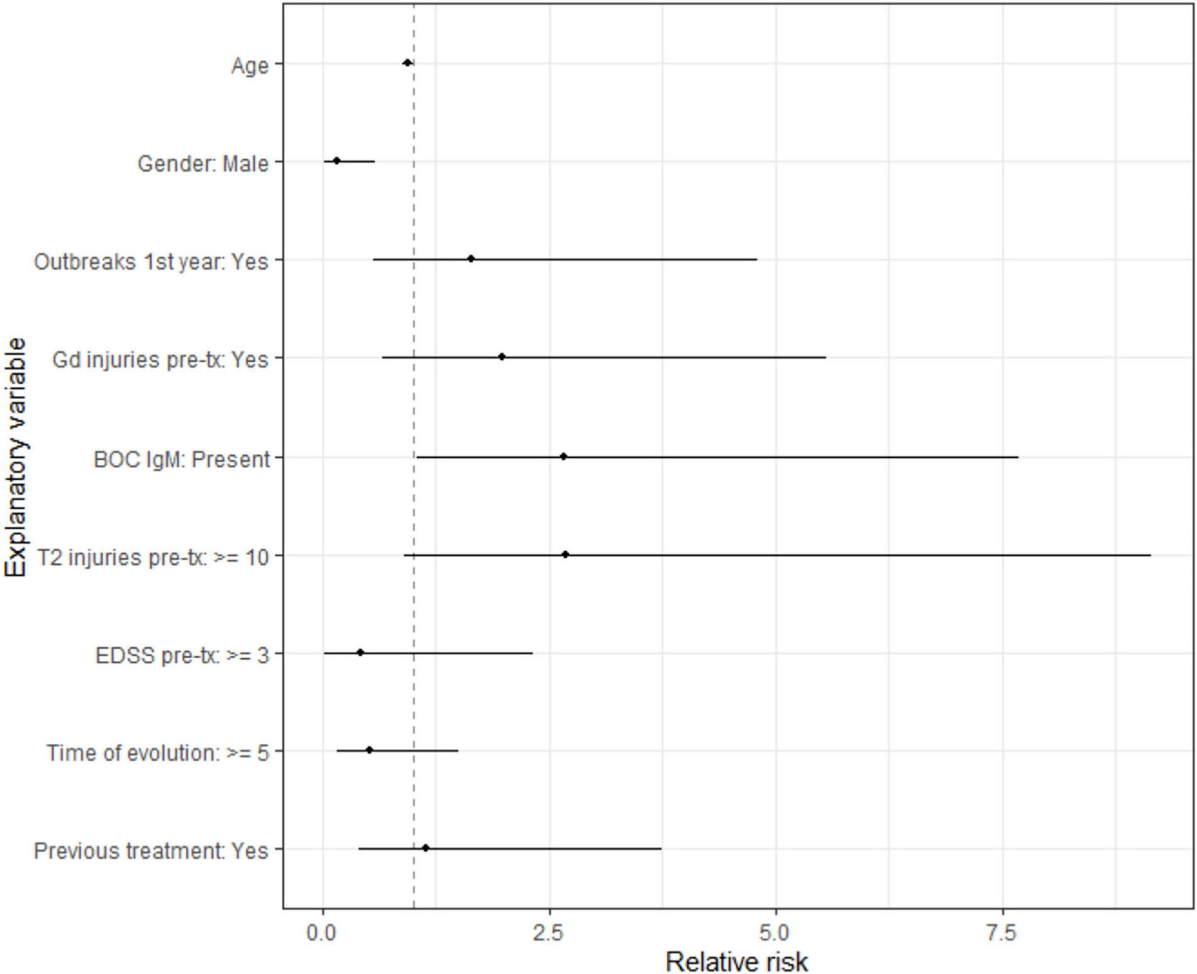
Relapses relative risk according to patient traits.

We evaluated the number of cumulative relapses that patients presented during treatment based on their demographic and clinical characteristics using Poisson regression. It is observed that the risk of relapses is 6 times higher (≈1/0.1588) in women than in men and that the risk of relapses is approximately 2.5 times higher in patients with BOG IgM present compared to those without BOC IgM (Supplementary Table 2 and Figure 5).

**Figure 6:**
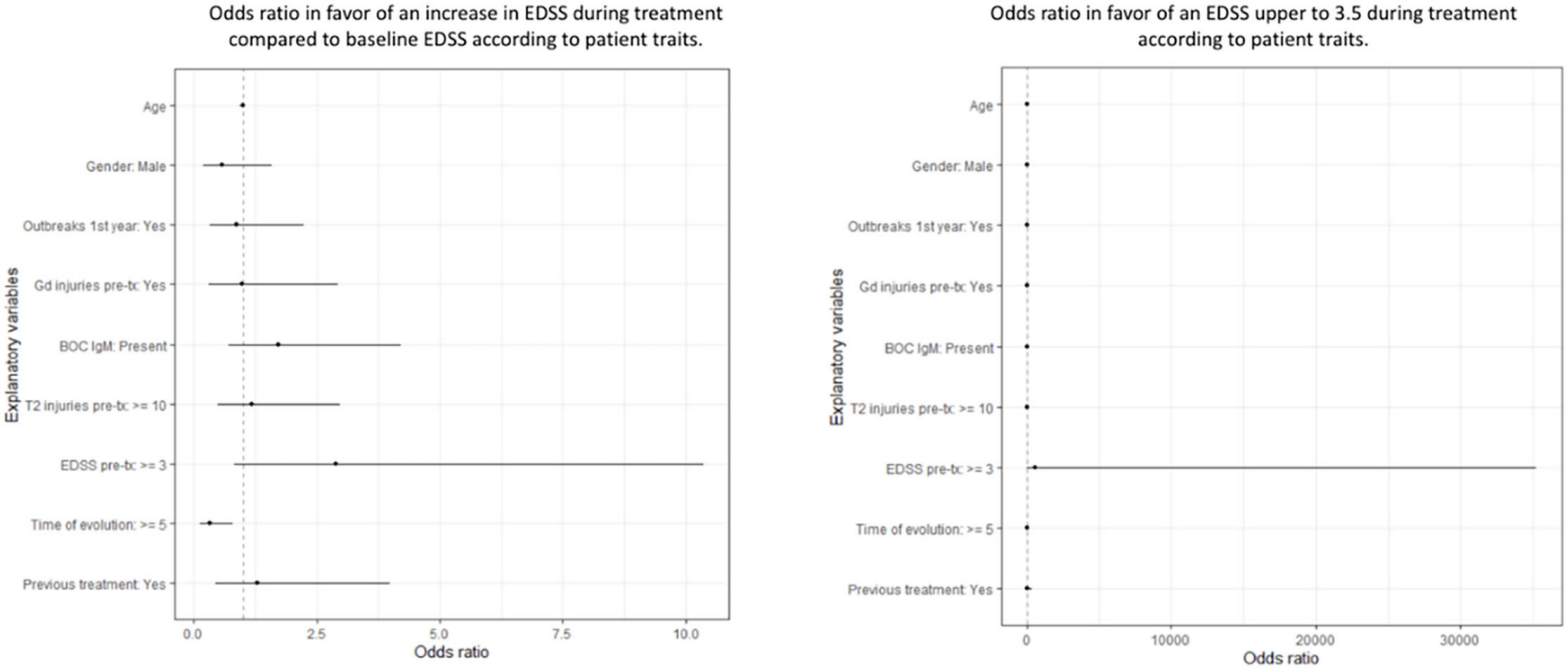
Odds ratio in favor of an increase in EDSS during treatment compared to baseline and odds ratio in favor of an EDSS upper to 3.5 during treatment according to patient traits.

We applied a logistic regression model to evaluate the risk of an increase in EDSS score during treatment with respect to baseline, and the model did not show any significant association with any of the variables evaluated (Supplementary Table 3 and Figure 6). Likewise, we have observed that the probability of presenting an EDSS score ≥3.5 during treatment is higher in those patients who presented EDSS scores ≥3 before starting treatment with teriflunomide (Supplementary Table 4).

**Figure 7:**
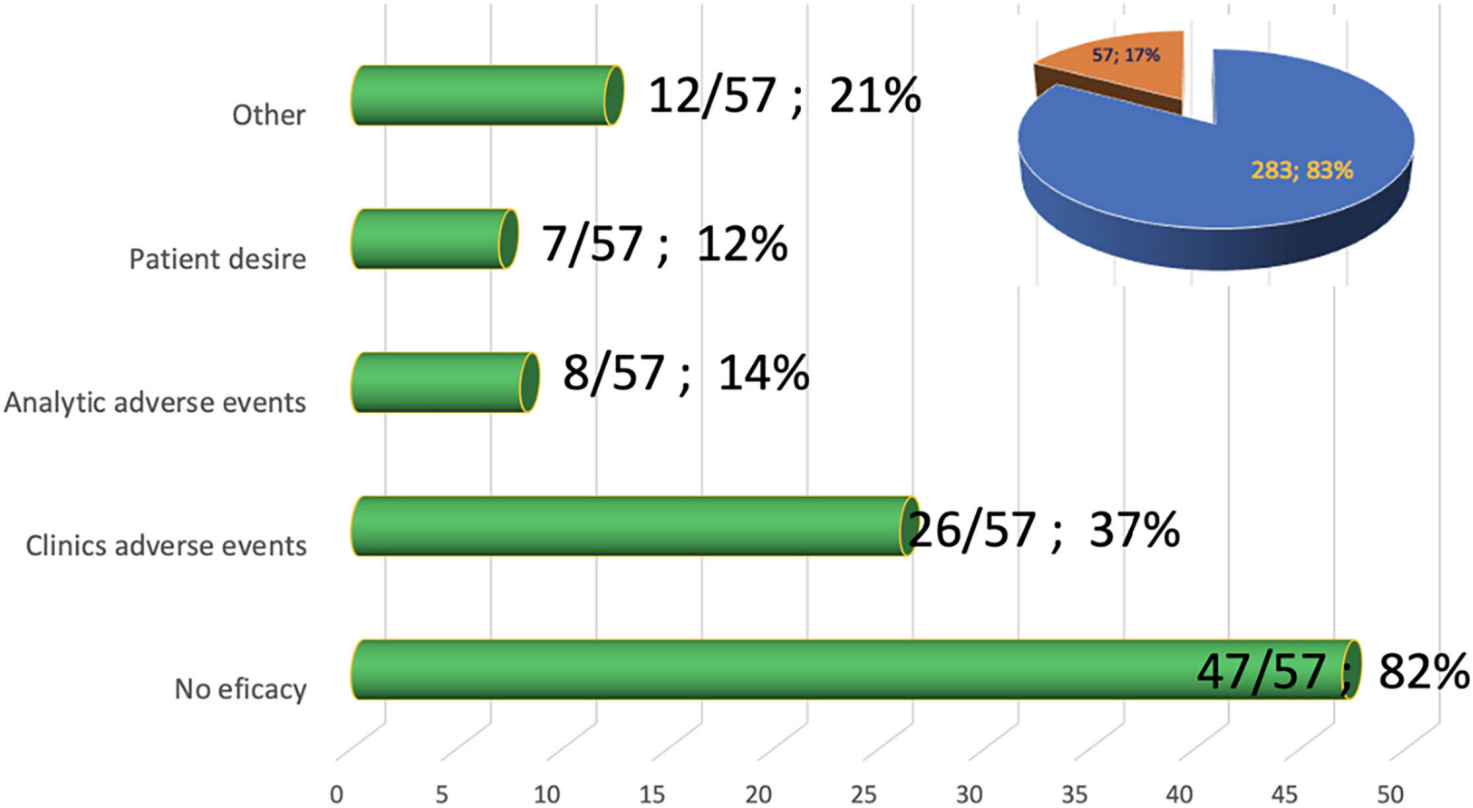
Causes of withdrawal from Teriflunomide treatment. Sector diagram shows the % of patients still being treated with Teriflunomide (blue).

Regarding the duration of follow-up, the dropout rate, and persistence with treatment: 236 patients were followed-up with for at least 12 months, 162 for 24 months, 54 for 36 months, and 8 patients with a minimum follow-up of 48 months, for a total of 754 patients/year. No statistically significant relationship is observed between the characteristics of the patients and the probability of continuation or not with treatment. The persistence at the end of the study was 83%: 282 patients were still being treated with teriflunomide, and in the remaining 17% (57 patients) treatment had been withdrawn. The reasons for this withdrawal were as follows: 47 (82%) due to ineffectiveness at the clinician's criteria, 26 (37%) due to clinical adverse events, 8 (14%) due to analytical adverse effects, 7 (12%) due to patient's desire, and 12 (21%) for other reasons, among which would be pregnancy planning (Figure 7).

**Figure 8:**
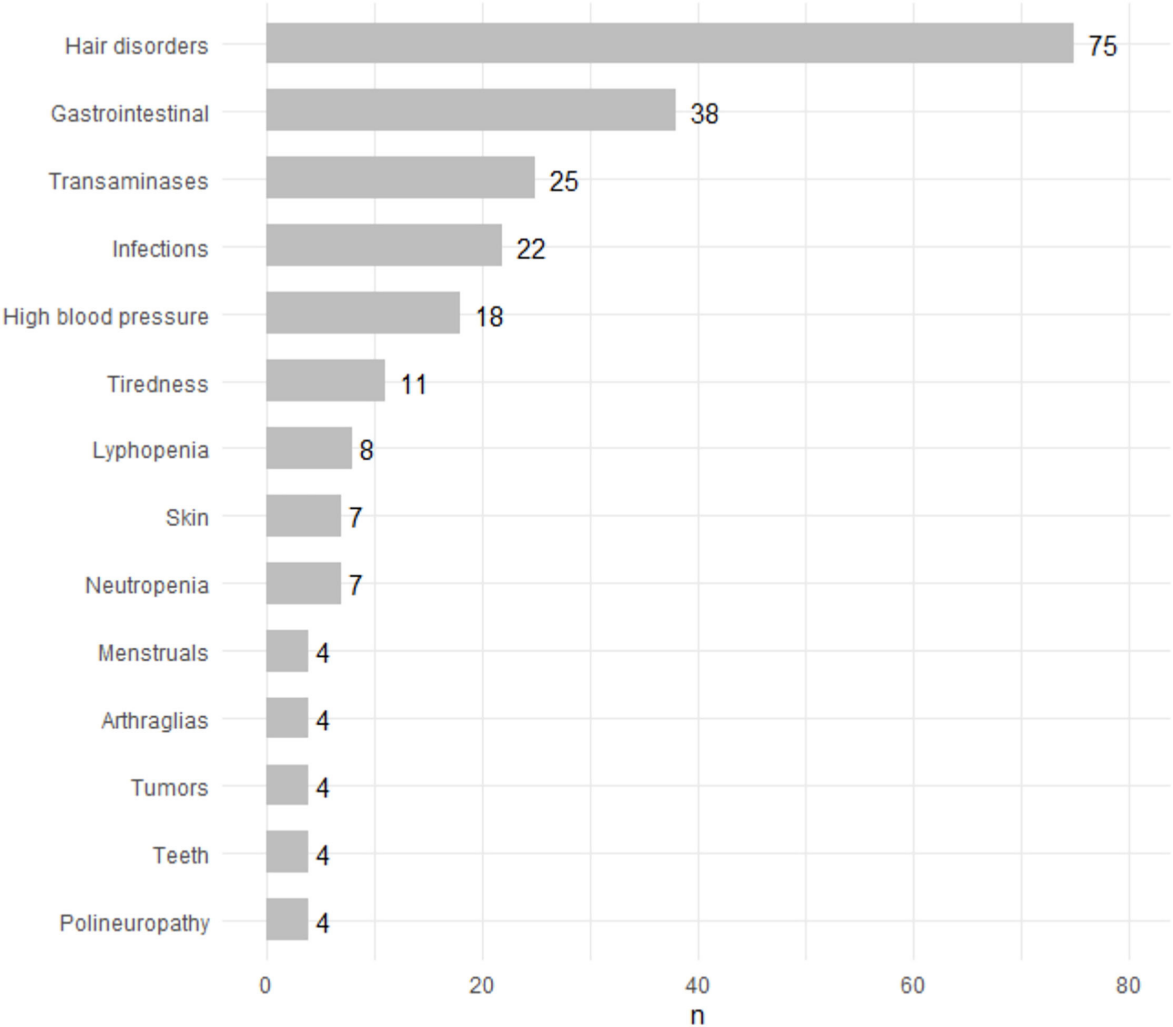
Adverse events.

As can be seen in Figure 8 and Table 3, the most frequently reported adverse event was hair alterations in 70 patients (20.5%), although only for 3 of them was it the reason for withdrawal. Other adverse events are as follows: gastrointestinal disorders in 51 patients (15%), and only for 6 of them was it the cause of discontinuation of the drug; 33 patients suffered infections (9.7%), of which 4 were the cause of withdrawal i. An increase in blood pressure was reported in 16 patients (4.7%), and only 1 patient discontinued therapy for this reason. There were 7 cases of peripheral nervous system involvement, and in all of them, the treatment was withdrawn although they were mild clinical pictures with only sensory symptoms. There were 5 (1.47%) patients who suffered from urticariform symptoms. Other symptoms, such as arthralgias, menstrual disorders, and alterations in the dentition have also been described: in no of them was the drug withdrawn by these adverse events.

**Table 3:** Adverse events, prevalence, and reason for withdrawal.

**Adverse events**	***N* (%)**	**Reason for withdrawal (%)**
Hair alterations	70 (20.5%)	3 (0.88%)
Gastrointestinal disorders	51 (15%)	6 (1.76%)
Infections	33 (9.7%)	4 (1.17%)
High Blood pressure	16 (4.70%)	1 (0.29%)
Weariness	8 (2.35%)	0
Polyneuropathy	7 (2.05%)	7 (2.05%)
Skin disorders	5 (1.47%)	3 (0.88%)
Arthralgias	4 (1.17%)	0
Teething disorders	4 (1.17%)	0
Menstrual disorders	3 (0.88%)	0
Tumors	4 (1.17%)	3 (0.88%)
Benign ovary tumor	1 (0.29%)	0
Ovarian neoplasm	1 (0.29%)	1 (0.29%)
Breast neoplasia	1 (0.29%)	1 (0.29%)
Glioblastoma	1 (0.29%)	1 (0.29%)
High transaminases	32 (9.41%)	10 (2.94%)
Neutropenia	7 (2.05%)	2 (0.58%)
Linfopenia	8 (2.35%)	1 (0.29%)
Trombopenia	2 (0.58%)	0

From the laboratory point of view, elevation of transaminases has been observed in 32 patients (9.41%) but only in 10 of them was this the reason for withdrawal of the drug. Neutropenia was observed in 7 patients (2.05%), lymphopenia in 8 patients, and thrombopenia in 2 patients have also been described. All of these cases have been mild and reversible without the need to modify the treatment.

We have described 4 patients with tumors, one of them a benign ovarian tumor. The other 3 were causes of withdrawal: there were an ovarian neoplasm, a breast neoplasm, and a malign glioma.

## Discussion

This is an observational study of real clinical practice that analyzes patients diagnosed with RRMS who have been treated with teriflunomide in 7 units and monographic clinics of demyelinating diseases. Real clinical practice studies provide us with data on the efficacy of different treatments in the medium and long term, safety beyond clinical trials, and can help us to know the patient profile appropriate for each therapy; especially now when we have a great number of therapies to be used in RRMS patients.

We have also put this data in context, in relation to other large cohorts of patients treated with teriflunomide, specifically with the Danish registry (4), the German registry TAURUS-MS (5), and the Italian registry (6).

The patients have a mean age of 46 years and multiple sclerosis of an average of 11.5 years of evolution. 19.53% of the patients are without previous therapies and the rest had been treated before, most with platform therapies. Almost 20% of the patients were naïve; this data may suggest a selection bias in favor of a more benign population; however, it should be noted that this group includes patients who previously refused injectable therapies and others who were treated in the past, but who did not receive treatment in the 6 months prior to starting teriflunomide.

It should be noted that of patients who initiated teriflunomide, only 10.03% did so due to the ineffectiveness of previous therapy, meaning that a significant proportion of them are patients with a stable and well-controlled disease with a previous drug. This partly explains why 64.31% of patients did not have relapses in the year prior to the onset of teriflunomide. Of the patients who did have prior relapses, 32.14% had one relapse in the previous year and 3.54% had two relapses or more. The mean EDSS score prior to diagnosis was 1.9, an expected value in patients in the first line of treatment.

The mean number of gadolinium-enhancing lesions per patient and per scan was 1.07 lesions at the beginning of teriflunomide treatment. The values referring to lesions in T2 sequences may lead to biases derived from the method of accounting for lesions, which is different in each center (Semi-automatic or manual methods performed by radiologists or by the neurologists themselves. The MRI protocols used in participants centers may be different), but we can say that practically 50% of the patients had 10 or more lesions in T2 sequences. The analysis of the T2-MRI may be one of the main biases of our study, and as we are aware of this, we did not want to include this variable as a marker of effectiveness nor have we wanted to develop composite variables (NEDA for example) given the poor consistency of this data.

Another highlight of the study and of the cohort studied is the high number of patients for whom a CSF study is available. In 262 patients we have the results of IgG oligoclonal bands. And in 147 of them, we know the profile of the IgM bands in the CSF, linked to forms of disease with worse prognosis (7).

Measuring the efficacy of treatment in the first 3 years, we have seen a reduction in the annual relapse rate and the cumulative number of relapses, compared to the pre-treatment year, with statistical significance, and despite the high proportion of patients who arrived at Teriflunomide with stabilized disease. An additional benefit measured by relapses is still achieved in this population. When we study the EDSS scores, it is observed that there is no increase throughout the follow-up; it remains stabilized. We have also seen a reduction in the presence of gadolinium-enhancing lesions with respect to the pre-teriflunomide situation. This reduction is statistically significant in the first and second years post-therapy.

From the analyzed data it is seen that patients under 40 years old have more relapses from the third year of treatment. This phenomenon is not seen in those over 40 years of age; the response to treatment may be longer-lasting in those over the age of 40. Likewise, the response is also better in the first year in patients who did not present relapses in the previous year and in those patients with previous therapy, which is quite logical and reinforces the idea that the change of therapy after another first-line drug for reasons of tolerability or comfort would be a risk to the good control of the disease. The benefits of the drug are similar in those patients with a disease of more or <5 years of evolution and in those with an EDSS score > or <3. We have not found an influence on the relapses from the fact of carrying IgG oligoclonal bands or not, however there are differences in those patients with IgM oligoclonal Bands, who responded worse to the treatment. It also appears that there was a worse response in the first year of treatment in those patients who had gadolinium-enhancing lesions on MRI in the study prior to the beginning of teriflunomide.

Our results are very similar to those in Germany and Italy. In all three studies, the high age of patients (around 46 years), the long time of evolution of MS (around 12 years), and a previous annual rate of relapses (around 0.6) are striking. The previous EDSS scores are more variable, from 1.9 in our series vs 2.9 in the Italian study. The other aspect to take into account is the high percentage of patients who took previous therapy, with 71.5% in our study and 75.2% in the German study. Despite this, teriflunomide achieves an added benefit over the annual relapse rate and a stabilization of EDSS scores, as seen in our registry and in the other revised European registries.

We have observed that the risk of relapses throughout follow-ups (number of relapses accumulated throughout follow-up) is about 6 times higher in women than in men. Similarly, this risk is about 2.5 times higher in patients with IgM oligoclonal bands.

After applying this multiple linear regression model, we have seen that the risk of suffering an increase in EDSS score throughout the follow-up was lower in those patients with a disease of 5 or more years of evolution, and the risk of an EDSS score ≥3.5 is higher in those patients with previous EDSS scores ≥3.

This analysis identifies a profile of a teriflunomide responder: a male patient with absent oligoclonal IgM bands, an EDSS score of <3, and more than 5 years of disease evolution. The Italian registry identified patients at risk of worse response to teriflunomide as those with a previous EDSS score of >4, if the EDSS score is between 2 and 4 and the patient has taken at least one previous treatment, or if the EDSS score is <2 and the patient has taken at least two previous treatments.

Regarding adverse events, our data are aligned with results observed in the pivotal Teriflunomide trials. Highlight an adverse event not reflected among the most frequent: teething disorders, which we have observed in a 1.5%.

The persistence rate was 83%, 10 points above observed in TEMSO trials, even taking into account the longer duration of our follow-up. We consider this variable important as it is the result of the efficacy, tolerability, safety, and convenience of teriflunomide. In our series, the persistence is the highest of the revised series (83%, compared to 78.5% of the German registry and 71% of the Italian registry).

In conclusion, our data indicate that teriflunomide is an effective and safe drug in the treatment of moderately active RRMS. It would be a good option in naïve patients or after a first poorly tolerated platform treatment. The responding patient could be a male with absent oligoclonal IgM bands, an EDSS score of <3, and <5 years of disease evolution.

## Data Availability Statement

The raw data supporting the conclusions of this article will be made available by the authors, without undue reservation.

## Ethics Statement

Ethical review and approval was not required for the study on human participants in accordance with the local legislation and institutional requirements. Written informed consent for participation was not required for this study in accordance with the national legislation and the institutional requirements.

## Author Contributions

LL is the main author of the manuscript. FP-M, SG, AB, FG, JD, MC-G, CQ-B, LN, LG, and BC are collaborators via contribution with patient date of each hospital. All authors contributed to the article and approved the submitted version.

## Conflict of Interest

The authors declare that the research was conducted in the absence of any commercial or financial relationships that could be construed as a potential conflict of interest.

## Publisher's Note

All claims expressed in this article are solely those of the authors and do not necessarily represent those of their affiliated organizations, or those of the publisher, the editors and the reviewers. Any product that may be evaluated in this article, or claim that may be made by its manufacturer, is not guaranteed or endorsed by the publisher.
