# Supplementary figures and images for: Treatment of Multiple Sclerosis With Teriflunomide. Multicenter Study of Real Clinical Practice in the Valencian Community-Spain
Source: Front Neurol. 2021 Oct 29;12:727586. doi: 10.3389/fneur.2021.727586 (PMC8603659; doi:10.3389/fneur.2021.727586)

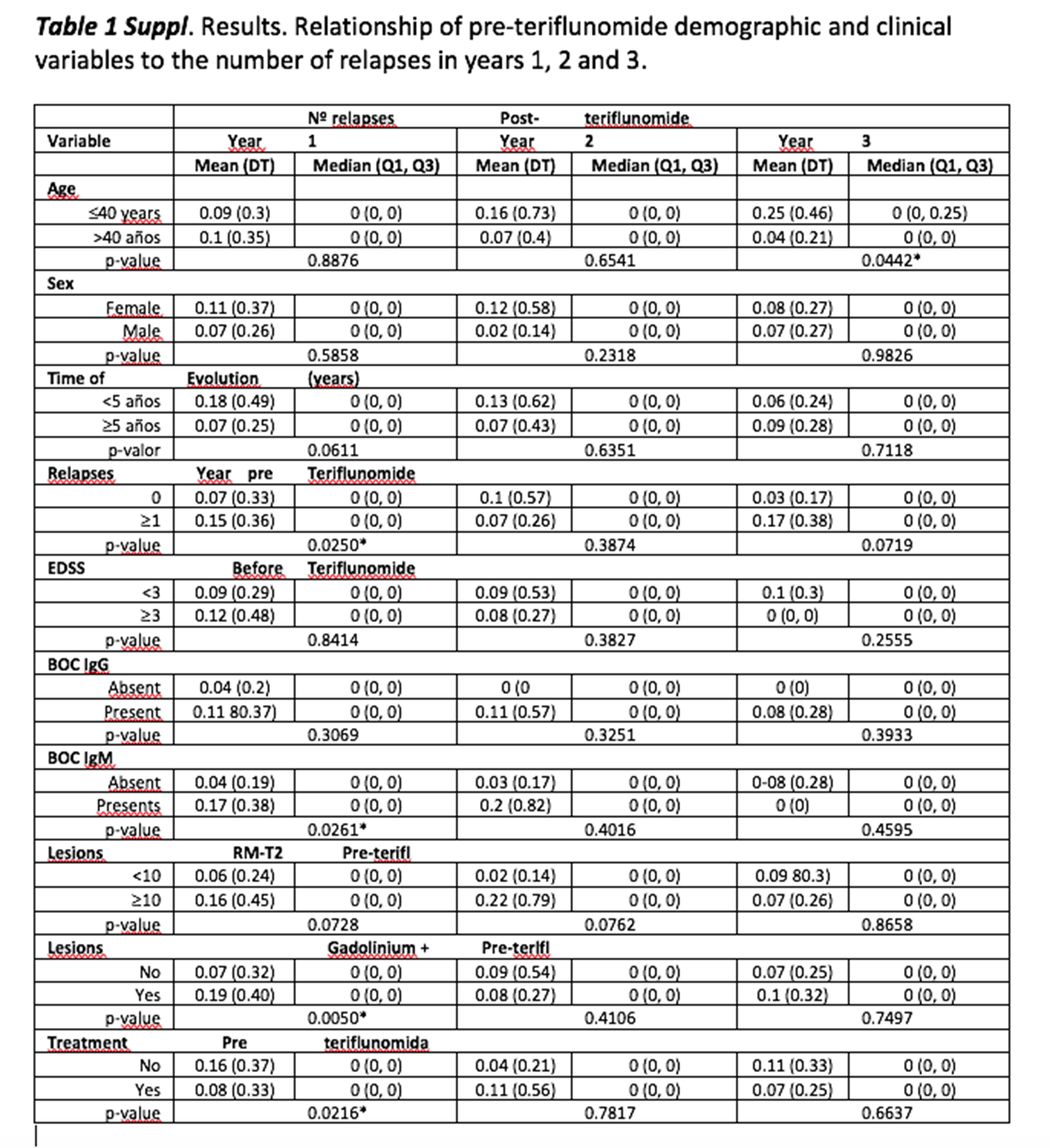

Supplement: Supplementary file 1 [file Image_1.png]

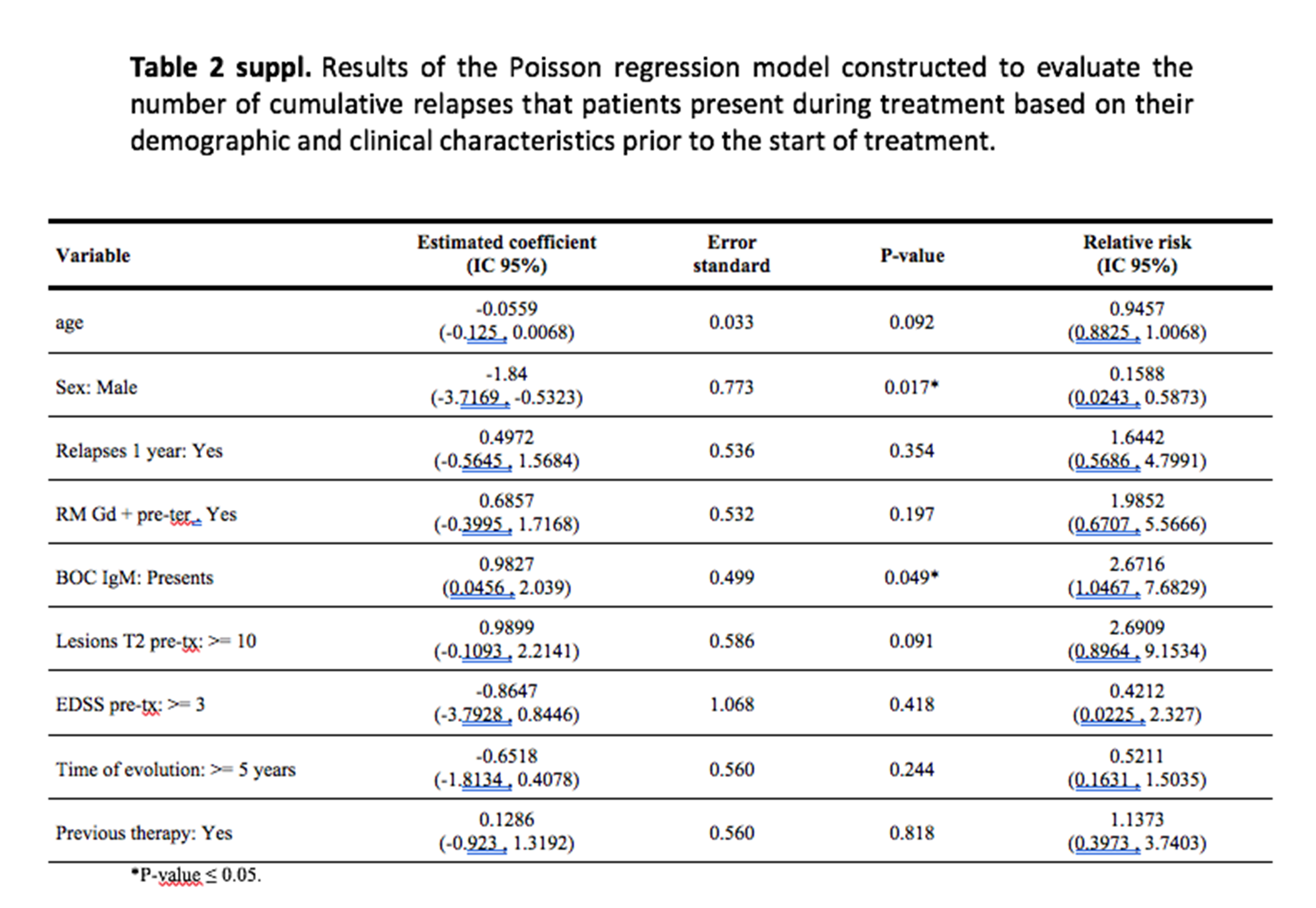

Supplement: Supplementary file 2 [file Image_2.png]

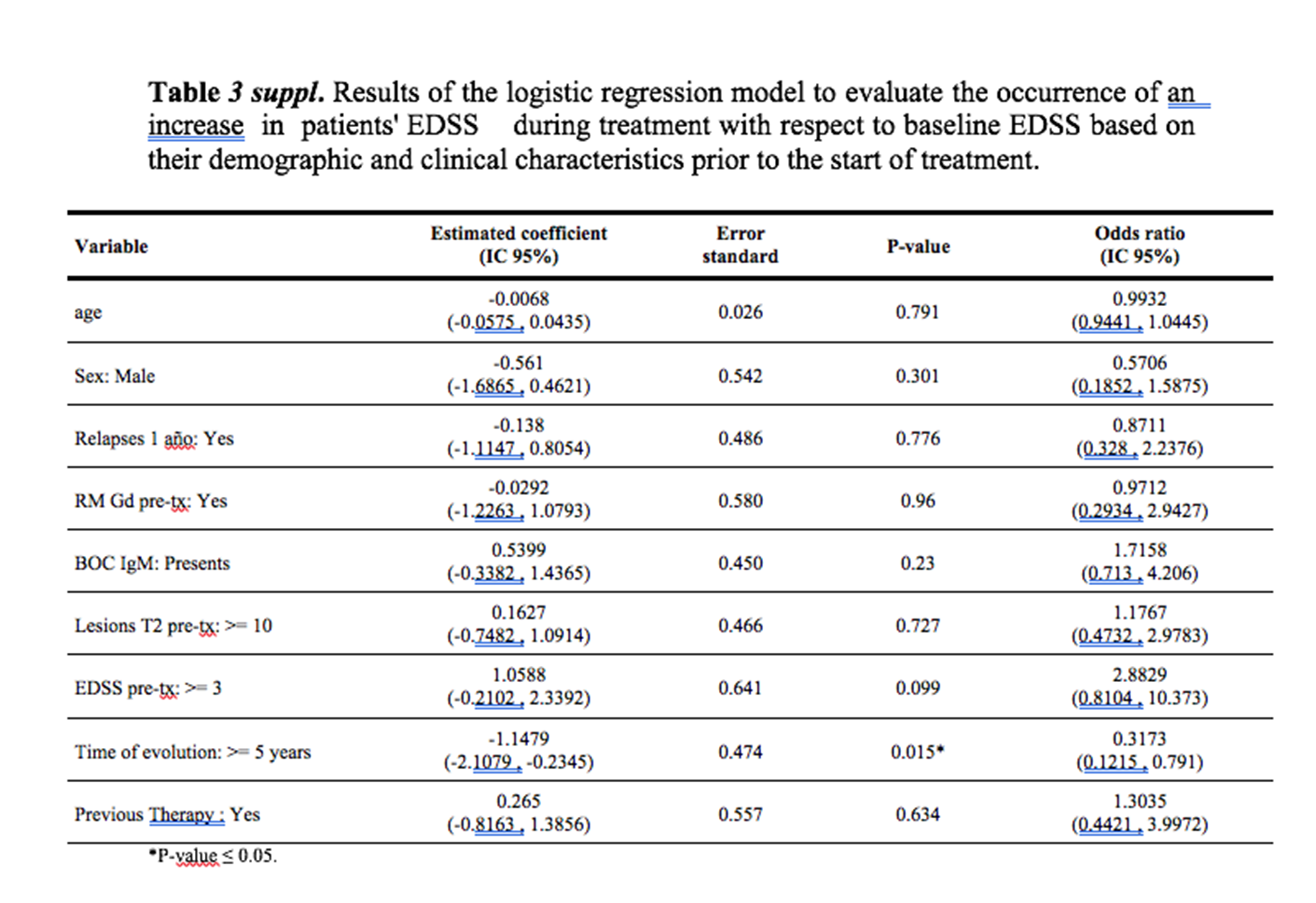

Supplement: Supplementary file 3 [file Image_3.png]

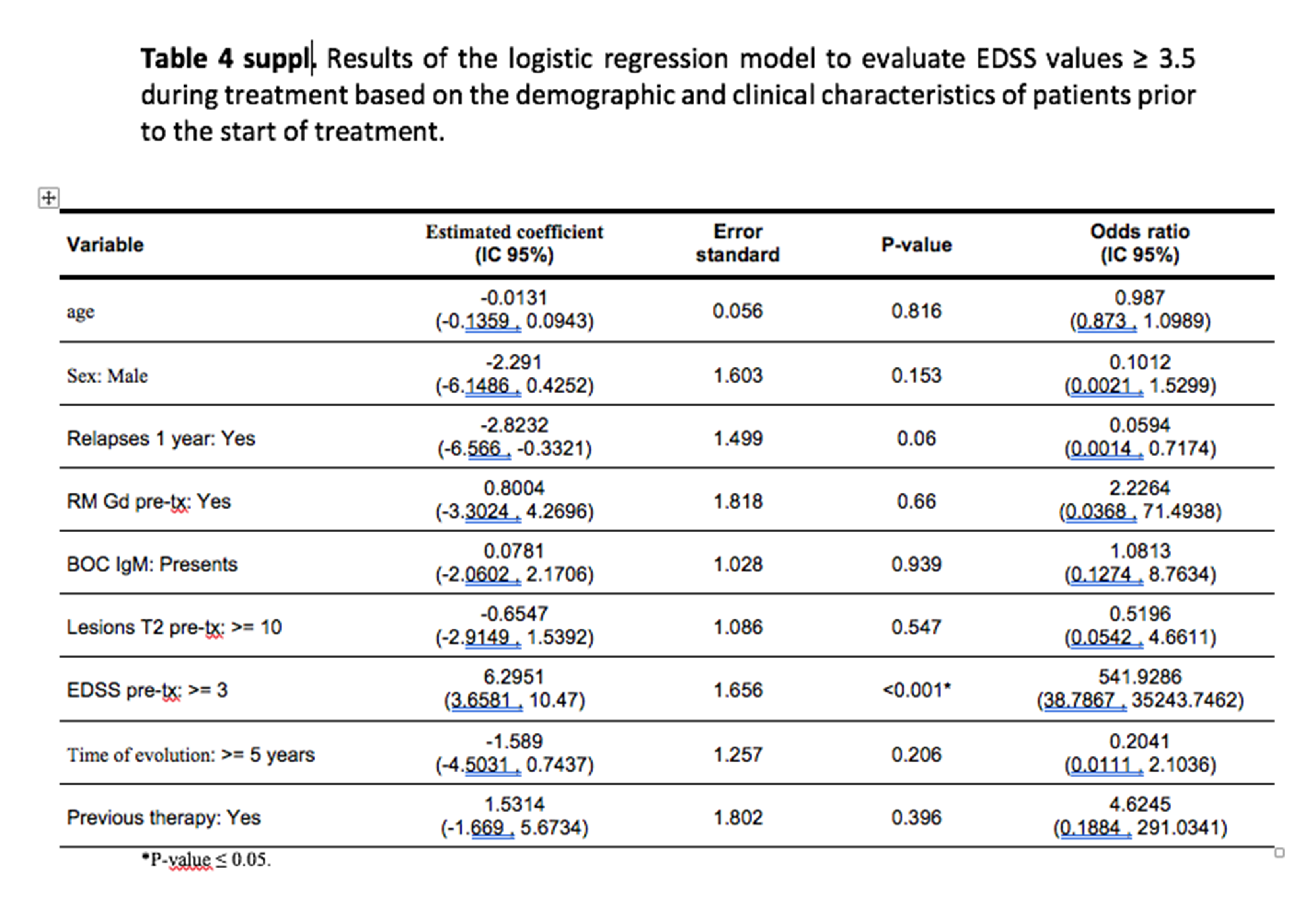

Supplement: Supplementary file 4 [file Image_4.png]
